# Overexpression of NELFE contributes to gastric cancer progression via Wnt/β-catenin signaling-mediated activation of CSNK2B expression

**DOI:** 10.1186/s13046-021-01848-3

**Published:** 2021-02-01

**Authors:** Shijun Yu, Li Li, Hui Cai, Bin He, Yong Gao, Yandong Li

**Affiliations:** 1 Department of Oncology, Shanghai East Hospital, Tongji University School of Medicine, Shanghai, 200120 China; 2 Department of Geriatrics, The Second Affiliated Hospital of Zhejiang University, Hangzhou, 310009 China

**Keywords:** NELFE, Wnt/β-catenin, CSNK2B, Gastric cancer, Tumorigenesis

## Abstract

**Background:**

Accumulating evidence has highlighted the importance of negative elongation factor complex member E (NELFE) in tumorigenesis. However, the relationship between NELFE and gastric cancer (GC) remains unclear. This study aimed to explore the expression pattern and specific function of NELFE in GC.

**Methods:**

NELFE expression was evaluated by immunohistochemistry and qRT-PCR in GC tissues, respectively. Cell proliferation, migration and invasion were measured by CCK-8, colony formation, transwell assays, and nude mice model. Bioinformatics analysis was performed to search potential target genes of NELFE, and a Cignal Finder 10-Pathway Reporter Array was used to explore potential signaling pathways regulated by NELFE. Dual-luciferase reporter assays, qRT-PCR and western blotting were conducted to verify their regulatory relationship. The expression correlations among NELFE, β-catenin and CSNK2B were further explored by immunohistochemistry on consecutive resections.

**Results:**

NELFE was significantly overexpressed in GC tissues both in protein and mRNA level and negatively correlated with the prognosis of GC patients. Gain- and loss-of-function experiments showed that NELFE potentiated GC cell proliferation and metastasis in vitro and in vivo. CSNK2B was identified as a downstream effector of NELFE. Wnt/β-catenin signaling may mediate the regulation of CSNK2B by NELFE. In addition, NELFE, β-catenin and CSNK2B were all remarkably upregulated in tumor tissues compared with adjacent normal tissues, and their expression levels in GC were positively correlated with each other.

**Conclusion:**

Our findings reveal a new NELFE-Wnt/β-catenin-CSNK2B axis to promote GC progression and provide new candidate targets against this disease.

**Supplementary Information:**

The online version contains supplementary material available at 10.1186/s13046-021-01848-3.

## Background

Dynamic regulation of transcription elongation shortly after initiation by RNA polymerase II (RNAPII) plays key roles in the implementation of gene expression widespread in metazoans [1, 2]. This step is modulated by positive and negative transcription elongation factors which are known as P-TEF and N-TEF, respectively [3, 4]. Acting as N-TEFs, the proteins negative elongation factor (NELF) and 5,6-dichloro-1β-D-ribofuranosylbenzimidazole (DRB)-sensitivity-inducing factor (DSIF) cooperatively inhibit elongation by associating with the RNAPII complex, and this effect can be alleviated by P-TEFb-mediated phosphorylation of RNAPII, DSIF and NELF, thus continuing gene transcription [4, 5]. Dysregulation of the elongation step has been found to contribute to human diseases including cancer [6, 7].

NELF has been shown the essential roles in the regulation of promoter-proximal pausing of RNAPII [8, 9]. It includes four subunits, NELFA, B, C/D, and E, and only NELFE (also known as RDBP) has a RNA binding ability. The structure of NELFE comprises of an N-terminal leucine zipper motif, a central domain abundant in Arg-Asp dipeptide repeats (the RD motif) and a C-terminal RNA recognition motif (RNM) containing two highly conserved elements dubbed RNP1 and RNP2, respectively [10]. It has been implicated that NELFE binds to a set of RNA sequences and contributes to the activities of NELF [10, 11]. More importantly, NELFE has been found to be involved in cancer progression in several studies. For instance, oncogenic activation of NELFE promotes hepatocellular carcinoma (HCC) cell proliferation and predicts the strong metastatic potential of HCC cells [12], whereas knockdown of NELFE results in enhanced cell colony growth of breast cancer cells when treated with estrogen-like reagents [13]. While these findings implicate a critical role of NELFE in cancers, its specific functions and underlying molecular mechanisms are not fully understood.

Gastric cancer (GC) is one of the most lethal human malignancies worldwide [14]. Although advances of modern medical science have improved the survival rates of patients with GC, there is still a dearth of effective biomarkers and effective treatments, which represents a major challenge in the management of GC. In the present study, we focused on NELFE and investigated its role in the development of GC and explore the underlying molecular mechanisms. We showed that NELFE was overexpressed in GC tissues and could predict poor prognosis. The upregulation of NELFE facilitated GC cell proliferation and metastasis in vitro and in vivo. CSNK2B was identified as a downstream effector of NELFE. Further mechanistic study revealed that Wnt/β-catenin signaling was involved in the regulation of CSNK2B by NELFE.

## Materials and methods

### Tissue samples and immunohistochemistry methods

To analyze the protein expression of NELFE in GC, 2 independent commercial tissue microarrays (TMA) containing 68 primary tumors, 53 adjacent normal tissues and 35 metastatic tumors from patients with GC were purchased from Shanghai Outdo Biotech Co., Ltd., Shanghai, China (#HStm-Ade076Met-01 and #HStm-Ade120lym-01). For further IHC analysis of NELFE, β-catenin and CSNK2B expression and their correlation in GC, consecutive sections of a TMA containing 75 paired GC tumor and adjacent normal tissues were purchased from Shanghai Outdo Biotech Co., Ltd., Shanghai, China (#HStmA150CS02). The clinical characteristics of the patients can be checked at the website: http://www.superchip.com.cn/biology/tissue.html. Standard immunohistochemical staining procedures were performed using a specific antibody against NELFE (#sc-32,912, Santa Cruz Biotechnology, USA) at a dilution of 1:200, and the immunostained sections were scanned using an Aperio ScanScope CS scanner (Aperio, Vista, USA) and visualized via Aperio ImageScope software (Aperio, Vista, USA). The results were analyzed by two pathologists blinded to the clinical information independently based on the percentage of stained cells and staining intensity. Briefly, the percentage of NELFE/β-catenin/CSNK2B-positive cells was classified into 5 groups: < 10% (0), 10–25% (1), 25–50% (2), 50–75 (3), and > 75% (4). The staining intensity was divided into 4 groups: no staining (0), light brown (1), brown (2), and dark brown (3). The overall IHC scores of NELFE/β-catenin/CSNK2B were calculated using the following formula: overall score = percentage score×intensity score. The samples with an overall score ≤6 were defined as weak staining, and > 6 were defined as strong staining. In addition, 28 paired of GC samples and adjacent non-cancer tissues were collected from patients who underwent surgical resection and signed an informed consent in Shanghai East Hospital. After resection, these GC tissue samples were immediately frozen in liquid nitrogen and stored at − 80 °C for RNA extraction. The use of human samples in this study was approved by the ethics committee of Shanghai East Hospital, Tongji University School of Medicine.

### Cell culture and reagents

The human GC cell lines AGS, MGC-803, HGC-27, SGC-7901 and BGC-823 were obtained from the Shanghai Cell Bank of the Chinese Academy of Sciences, Shanghai, China. Cells were maintained in Dulbecco’s modified Eagle’s medium (DMEM; Corning, Inc., Corning, NY, USA) supplemented with 10% fetal bovine serum (FBS) and 1% penicillin/streptomycin (M&C Gene Technology Ltd., Beijing, China). The cells were cultured under standard conditions in a humidified incubator with 5%CO_2_. To activate Wnt signaling, the indicated cells were treated with 5 μM Wnt agonist 1 [15] (#S8178, Selleck Chemicals, Shanghai, China) for 24 h.

### Plasmid construction, RNA inference and lentivirus transduction

A full-length cDNA encoding NELFE gene was PCR-amplified cloned into pcDNA 3.1 mammalian expression vector (pNELFE), and empty pcDNA 3.1 vector (pVEC) was used as control group. For transient expression of NELFE, cells were seeded into a 6-well plate at 80% confluence and were transfected with 2.5 μg of pNELFE or pVEC using Lipofectamine 3000 (Invitrogen, Thermo Fisher Scientific, US) according to the manufacturer’s protocol. NELFE or CSNK2B-specific small inference RNAs (siRNAs) and negative control siRNA (siNC) were synthesized by GenePharma (Shanghai, China). The sequences are as follow: siNELFE-1: 5′-GGAAAGGGAAUACUCUCUAdTdT-3′; siNELFE-2: 5′-GAUUCCUUGUGCCUCAUAUdTdT-3′; siCSNK2B-1: 5′-CUCCGUGGCAAUGAAUUCUdTdT-3′; siCSNK2B-2: 5′-GUCAAGACGAUUCGCUGAUdTdT-3′; siNC: 5′-UUCUCCGAACGUGUCACGUdTdT-3′. For siRNA transfection, the indicated cells were seeded into a 6-well plate with a confluence of 20–40%, and the siRNAs were transfected into these cells using Lipofectamine 3000 (Invitrogen, Thermo Fisher Scientific, US) as per the manufacturer’s instructions. NELFE-knockdown lentivirus (shNELFE) was packaged (siNELFE-1 sequence was used) and supplied by Genepharma (Shanghai, China), the NELFE-overexpression lentivirus (LV-NELFE) was constructed and packaged by Tuzhu Biotech (Shanghai, China). For lentivirus transduction, the host cells at a confluence of 50% were transduced with the lentivirus in the presence of polybrene (8 μg/ml) and stably infected cells were selected with puromycin (2 μg/ml).

### Microarray data collection and bioinformatic analysis

The microarray data involved in this study were obtained from GEO datasets of NCBI (GEO accession number: GSE19826 and GSE13911), and the expression levels of CSNK2B were analyzed by GEO2R tool online. Kaplan-Meier survival curves were generated using Kaplan–Meier plotter (http://kmplot.com/analysis/) [16], a comprehensive online platform which is capable to assess the effect of over 54,000 genes (mRNA, miRNA, protein) on survival in 21 cancer types. The co-expressing genes of NELFE in GC were identified using the Coexpedia database (http://www.coexpedia.org/) [17], and their correlation was further verified by Gene Expression Profiling Interactive Analysis (GEPIA, http://gepia.cancer-pku.cn/) [18]. CSNK2B-interacting proteins and their KEGG pathway enrichments were analyzed by STRING database (Version 11.0, https://string-db.org/) [19]. The KEGG pathway map was generated using ggplot2 and Cario packages under R v3.5.2.

### Dual luciferase reporter assay

TCF/LEF1-Luc reporter plasmid was obtained from Genomeditech (Shanghai, China). For CSNK2B promoter-reporter plasmid construction, 1200 bp upstream promoter region of CSNK2B gene was cloned into pGL3 luciferase reporter vector. Dual luciferase reporter assays were carried out with a dual luciferase kit (Promega, Wisconsin, USA) as follows: Cells were seeded into a 24-well plate with a confluence of 70–90% prior to transfection. The indicated cells were co-transfected with the TCF/LEF1-Luc reporter plasmids or CSNK2B promoter-reporter plasmids and Renilla luciferase constructs using Lipofectamine 3000 as described above. After incubation for 24 h, cells were lysed using the dual luciferase kit and the luciferase activities were measured on a Glomax-multi Luminometer (Promega, Wisconsin, USA) according to the manufacturer’s protocols. Relative luciferase activity was calculated according to the following formula: luciferase activity = firefly luciferase bioluminescence/ Renilla luciferase bioluminescence.

### Pathway reporter array

Cignal™ Finder 10-Pathway Reporter Array (Qiagen, Hilden, Germany) was performed to concurrently assess whether NELFE influenced the activity of 10 different tumor-associated signaling pathways in accordance with the manufacturer’s specifications, and luciferase activities of different pathways were determined using a dual luciferase kit (Promega, Wisconsin, USA) as described above.

### Cell proliferation assay

The viability of GC cell lines was assessed using the Cell Counting Kit-8 (CCK-8) assay kit (Dojindo, Kumamoto, Japan). Briefly, cells were seeded into a 96-well plate at a density of 3000 cells per well suspended in 100 μl of complete culture medium. After incubation overnight, cells were treated with CCK-8 solution (10 μl per well) at the scheduled time intervals (0, 24, 48, 72, 96 h) and incubated for 1 h. Subsequently, the optical density (OD) values were measured at a wavelength of 450 nm on an automated microplate reader (SpectraMax M5, Molecular Devices, USA). For colony formation assay, cells were seeded into a 6-well plate with a density of 1000 cells/well in triplicate and cultured under normal conditions for 2–3 weeks. Colonies were fixed with 4% paraformaldehyde and stained with 0.5% crystal violet for 20 min, after which the plates were rinsed, dried, and colonies were photographed and counted using Image J software (v1.52, National Institutes of Health Freeware, USA).

### Cell migration assay

Migration assays were performed using a 24-well transwell chamber system (8 μm pore diameter, Costar #3422, Corning, USA) according to the protocol of manufacturers. The indicated cells suspended with 300 μl FBS-free medium were seeded into the upper chamber with a density of 30,000/well, while 600 μl medium containing 10% FBS was added into the lower chamber. After incubating for 24 h at 37 °C, the cells left in the upper surface were removed with a cotton swab, and the migrated cells on the lower surface were fixed with 4% paraformaldehyde and stained with 0.5% crystal violet for 30 min. Cells that migrated through the membranes were photographed and counted under an inverted microscope.

### Cell invasion assay

Cell invasion was measured with BD BioCoat Matrigel invasion chambers (BD Biosciences, #354480, USA). Cells suspended in 400 μl of serum-free DMEM were seeded into the upper chamber at a density of 5× 10^4^ cells/well, while 800 μl of DMEM containing 10% FBS was added to the lower chamber as chemoattractant. After incubation for 24 h at 37 °C, cells on the upper side were wiped off with a cotton swab, and invaded cells on the lower side of the membrane were fixed with 4% paraformaldehyde and stained with 0.5% crystal violet for 30 min. The cells were photographed and counted under an inverted microscope at × 100 magnification.

### Immunofluorescence assay

Expression and subcellular location of NELFE and CSNK2B were detected by immunofluorescence assay. The indicated cells were planted onto glass coverslips and cultured under normal conditions. After fixation with 4% paraformaldehyde and permeabilized with 0.1% NP40 in PBS, the coverslips were blocked with 50% horse serum in PBS for 1 h at 4 °C and subjected to incubation of anti-NELFE (1:50) and anti-CSNK2B (1:50) at 4 °C overnight. After incubated with fluorescein-conjugated secondary antibody (Invitrogen, USA) for 1 h, the coverslips were mounted with 4,6-Diamidino-2-Phenylindole (DAPI)-containing mounting medium (Sigma, USA). Immunofluorescence staining was visualized under a Leica SP8 confocal laser scanning microscope (Leica Biosystems, Germany).

### Western blot analysis

Total proteins were extracted from GC cell lines using RIPA lysis buffer (#20–188, Merck Millipore, MA, USA) with 1× protease and phosphatase inhibitor cocktails (Sigma-Aldrich, MO, USA). Total protein lysates were obtained by centrifuged at 4 °C and 12,000 rpm for 15 min, and protein concentration was determined by using a BCA Protein Assay Kit (#T9300A, Takara Bio, Kyoto, Japan). 4× SDS loading buffer (#KGP101X, Keygentec Inc., Shanghai, China) was added into the protein lysates and the samples were boiled for 5 min prior to electrophoresis. Aliquots of the proteins were separated by 10% SDS-PAGE and transferred onto a nitrocellulose membrane (0.45 μm, Bio-Rad, CA, USA), followed by block of non-specific binding with 5% non-fat milk in PBS containing 0.05% Tween-20 (PBST) for 1 h at room temperature. Then membranes were subjected to incubation with specific primary antibodies at 4 °C overnight and secondary antibodies at room temperature for 1 h (avoid light). Immunoblots were visualized by the Odyssey Digital Infrared Imaging System (LiCoR Biosciences, NE, USA), and β-actin was used for internal reference. Primary antibodies used in the present study are as follow: anti-NELFE (1:200, # sc-32,912, Santa Cruz Biotechnology, CA, USA), anti-CSNK2B (1:1000, # 20234–1-AP, Proteintech Group, Wuhan, China), anti-β-catenin (1:1500, #8480, Cell Signaling Technology, MA, USA) and anti-β-actin (1:5000, #60008–1-Ig, Proteintech Group, Wuhan, China). Goat anti-rabbit-DyLight 800 (1:1000, #SA5–35571, Thermo Fisher Scientific, MA, USA) and anti-mouse-DyLight 800 (1:1000, #SA5–35521, Thermo Fisher Scientific, MA, USA) served as secondary antibodies.

### Quantitative Real Time PCR (qRT-PCR) analysis

Total RNA was isolated using TRI Reagent (Sigma-Aldrich, MO, USA) and then reverse transcribed into cDNA by PrimeScript RT reagent Kit with gDNA Eraser (Takara Bio, Kyoto, Japan) according to the manufacturers’ protocols. Synthesized cDNA was subjected to qPCR analysis using TB Green Premix Ex Taq II (Takara Bio, Kyoto, Japan) on an ABI QuantStudio 6 Flex Real-time PCR system (Applied Biosystems, CA, USA) in accordance with the manufacturer’s instructions. β-actin was used as the internal reference gene and relative gene expression level was calculated using the comparative cycle threshold (ddCt) method. Primer sequences for qPCR analysis were synthesized HuaGene Biotech (Shanghai, China) and listed in Supplementary Table 1.

### Animal procedures

Four to six weeks male athymic BALB/c nude mice were obtained from SLAC Laboratory Animal Company (Shanghai, China) and were raised under pathogen-free conditions with access to distilled food and water. To establish a subcutaneous xenograft model, 2× 10^6^ of MGC-803/BGC-823 cells suspended in 100 μl of PBS were inoculated subcutaneously into left (LV-VEC/shNC) or right (LV-NELFE/shNELFE) flank axillary region of nude mice (5 mice per group). The size of tumors was measured every 7 days, and tumor volume was calculated using the following formula: Tumor volume (mm^3^) = length × width^2^/2. Tumor bearing mice were euthanatized on day 28 after cell injection, histologically intact tumors were removed, weighted and photographed. For the lung metastasis model, 3× 10^6^ the above-mentioned GC cells in 100 μl of PBS were injected intravenously into the nude mice (6 mice per group) through tail vein. On day 42, the nude mice were sacrificed, lung tissues were collected and the number of metastatic lung nodules was counted. The lung tissues were immersed in 4% paraformaldehyde for subsequent histological examination, and potential lung metastatic lesions were finally confirmed by hematoxylin and eosin (H&E) staining method. All animal experiments were approved by the Medical Ethics Committee of Shanghai East Hospital.

### Statistical analysis

All in vitro and in vivo experiments were performed independently at least three times in triplicate, and continuous variables were presented as mean ± standard deviation (SD). Statistical analysis was performed by using GraphPad Prism software 7.0. The student’s t-test or one-way analysis of variance (ANOVA) was used to compare the mean difference between binary or multiple variables, the χ2 test was used to explore the relationships between NELFE/β-catenin/CSNK2B expression and clinicopathological parameters of GC patients. Pearson correlation coefficient analysis was used for comparing the correlation among the expression of three genes. A *P* value of < 0.05 was considered statistically significant.

## Results

### NELFE is significantly upregulated in GC tissues

**Fig. 1:**
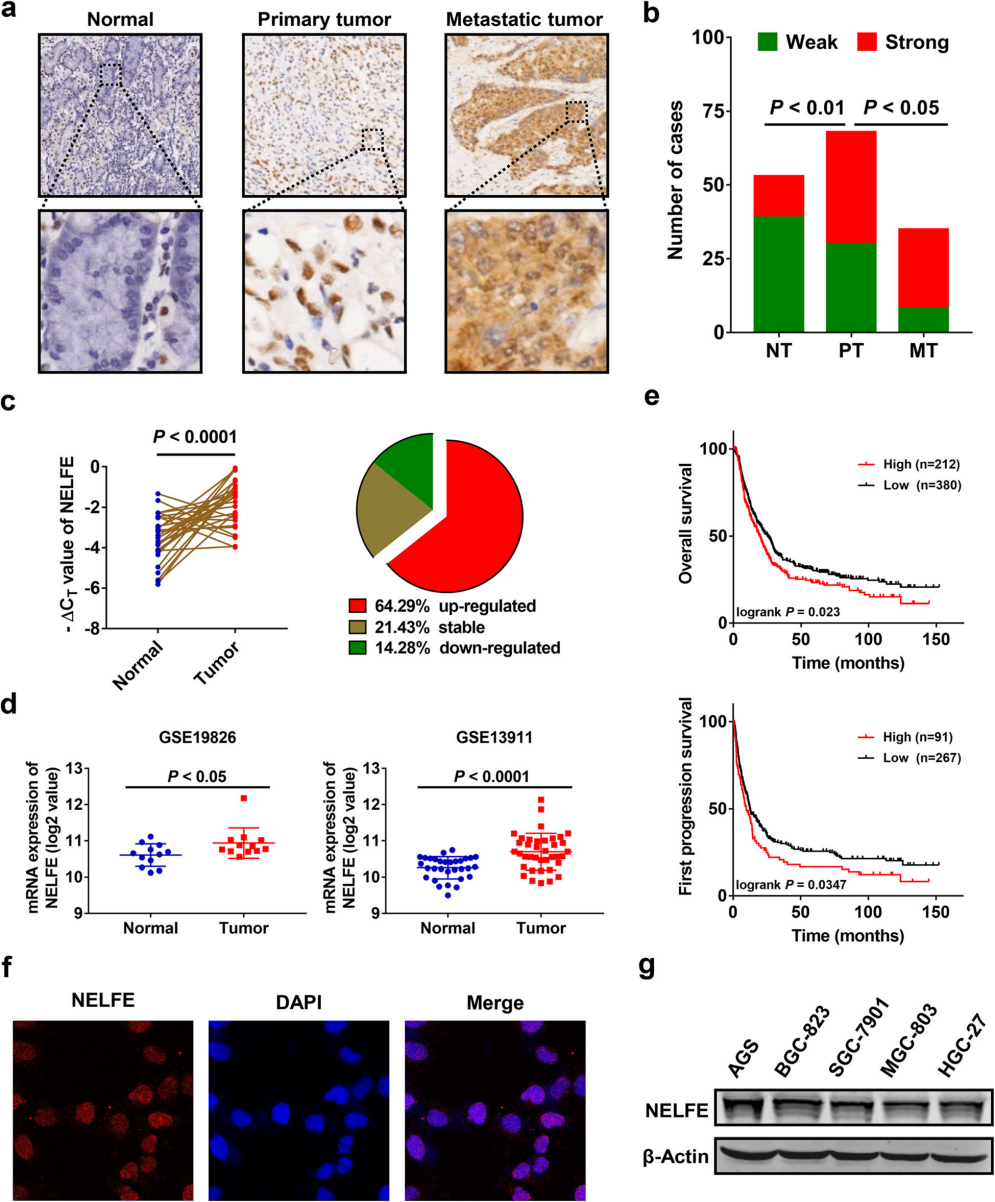
High NELFE expression predicts worse prognosis of patients with GC. **a** Representative IHC images of NELFE in adjacent normal tissues, primary tumor tissues and metastatic tumor tissues were shown. Magnification: 40× (upper) and 200× (bottom). **b** Statistical difference of NELFE protein expression among different sample types were analyzed using the Chi-square test. The samples with an IHC score ≤6 were defined as weak, and > 6 were defined as strong. **c** mRNA levels of NELFE in 28 paired tumor tissues and adjacent normal tissues were determined by qRT-PCR analysis, relative expression levels were represented as -ΔC_T_ values (left), and fold changes in tumor tissues relative to adjacent normal tissues were calculated to generate the pie chart (right). **d** Published NELFE gene expression data were obtained from the online GEO database at https://www.ncbi.nlm.nih.gov/geo/ (GEO accession number: GSE19826 and GSE13911). **e** Kaplan–Meier curves for overall survival and first progression survival analysis of NELFE in GC were generated using the Kaplan–Meier plotter database (http://kmplot.com/analysis). **f** Representative images of NELFE immunofluorescence staining (red) in MGC-803 cells. Magnification: 40×. **g** NELFE protein expression in different human GC cell lines by western blotting

To investigate the expression pattern of NELFE in GC, we performed immunohistochemistry (IHC) analysis using commercial tissue microarrays (TMAs) containing 68 primary gastric tumor tissues, 35 metastatic tumor tissues and 53 adjacent normal tissues derived from 102 GC patients, and representative IHC images of NELFE were shown in Fig. 1a. Compared with adjacent normal tissues, elevated NELFE expression was observed in both primary and metastatic GC tissues (Fig. 1b). Intriguingly, metastatic tumor tissues exhibited significantly higher NELFE expression than primary tumor tissues (*P* < 0.05, Fig. 1b), suggesting that NELFE might be a potential biomarker to distinguish metastatic gastric malignancies from primary gastric malignancies. Besides, additional tumor tissues and matched adjacent normal tissues from 28 GC patients were collected and subjected to quantitative real-time PCR (qRT-PCR) analysis. The result showed that the mRNA level of NELFE was also remarkably elevated in GC tissues (*P* < 0.0001, Fig. 1d), among which 18 cases (64.29%) exhibited an increase of more than 1.5-fold change (Fig. 1c). In order to confirm the reliability of above data, we analyzed the expression profiles of NELFE in human gastric cancer samples from public Gene Expression Omnibus (GEO) datasets (GEO accession number: GSE19826 and GSE13911). Consistently, NELFE expression in GC tumor samples was higher than that in normal tissues (Fig. 1d). To evaluate the prognostic significance of elevated NELFE expression in GC, survival analysis was conducted on an online Kaplan-Meier Plotter database using published microarray data from GC patients. As shown in Fig. 1e, high NELFE expression was negatively correlated with overall survival (*P* < 0.05) and first progression survival (*P* < 0.05) rates of GC patients. By immunofluorescence assay, we observed that the subcellular location of NELFE in MGC-803 cells was mostly in nucleus and partly in cytoplasm (Fig. 1f). In addition, the expression pattern of NELFE in various GC cell lines was shown in Fig. 1g. Taken together, these data suggested that NELFE is overexpressed in GC and may serve as a potential biomarker for the progression of GC.

### NELFE promotes GC cell proliferation in vitro and in vivo

**Fig. 2:**
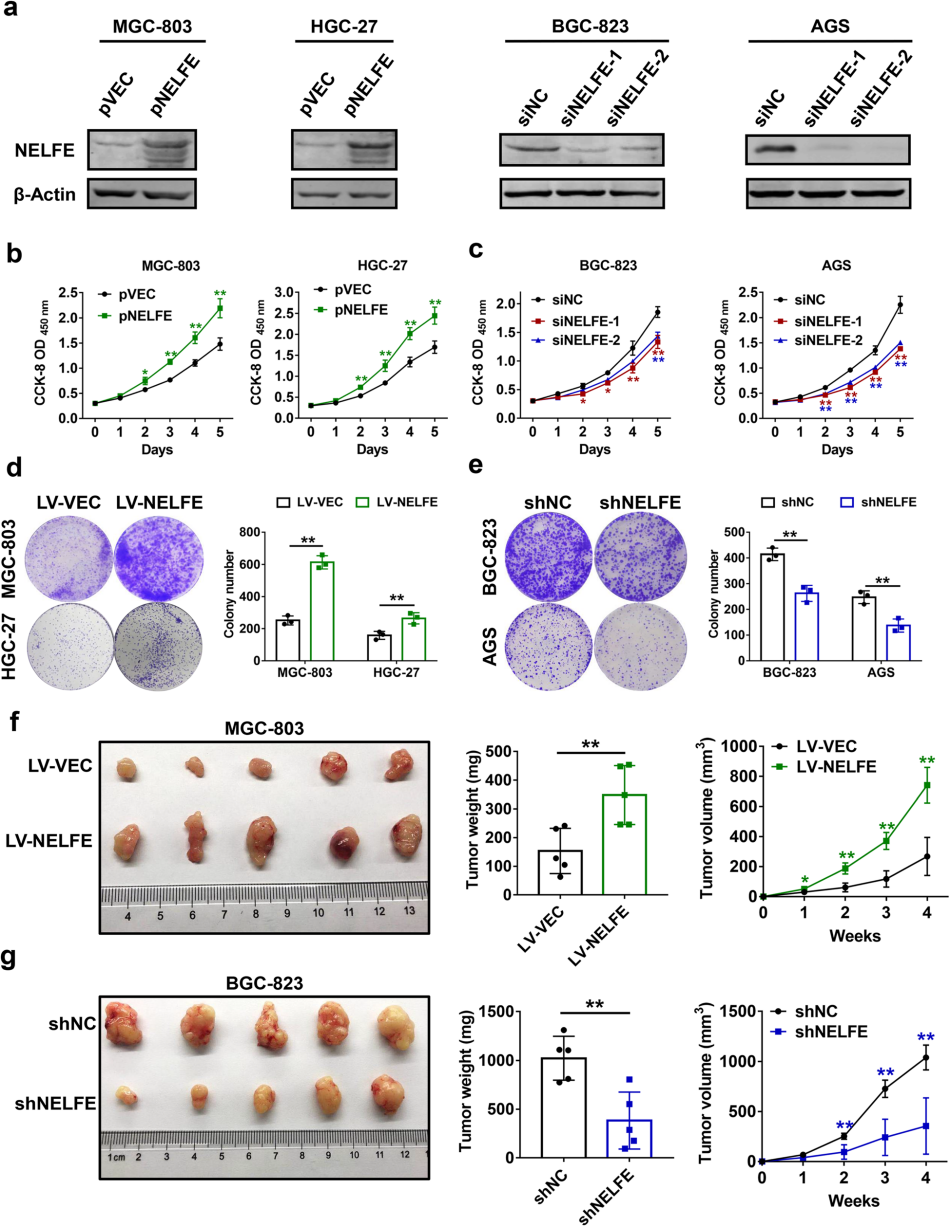
NELFE potentiates GC cell proliferation in vitro and in vivo. **a** NELFE-expressing plasmids or siRNAs against NELFE were transfected into the indicated GC cell lines, respectively. After 48 h, cells were lysed and western blot analyses were performed to verify the efficacies of overexpression and knockdown. **b-c** Cell viabilities were determined using CCK-8 cell proliferation assays at every 24 h after transfection of the above plasmids (**b**) or siRNAs (**c**). **d-e** Colony formation assays were conducted using the indicated GC cell lines with lentivirus-mediated stable overexpression (**d**) or knockdown (**e**) of NELFE. Representative colony images (left) and statistical analysis of the colony number in different groups (right) were shown. **f-g** Subcutaneous xenograf model of nude mice was established using MGC-803 or BGC-823 cells with stable overexpression (**f**) or knockdown (**g**) of NELFE (*n*=5 per group). Images of tumors (left), final tumor weight (middle) and tumor volume curve (right) were shown. *P < 0.05, **P < 0.01

We next assessed the biological functions of NELFE in GC. To achieve NELFE overexpression or knockdown in GC cell lines, NELFE-overexpression plasmid (pNELFE) was transfected into MGC-803 and HGC-27 cells with a relatively low NELFE expression, and specific siRNAs against NELFE (siNELFE-1 and siNELFE-2) were constructed and transfected into BGC-823 and AGS cells with high levels of NELFE expression, after which western blot analyses confirmed the transfection efficiencies (Fig. 2a). Subsequently, cell viability was measured using CCK-8 assays. The growth curves indicated that NELFE overexpression facilitated the proliferation of MGC-803 and HGC-27 cells (Fig. 2b), whereas BGC-823 and AGS cells with NELFE silencing exhibited reduced proliferation rates as compared to their control groups (Fig. 2c). The effects of NELFE on long-term proliferation abilities of GC cell lines were investigated using colony formation assays after the indicated cells were transduced with lentivirus for stable overexpression or silencing of NELFE. As expected, exogenous NELFE augmented colony formation of MGC-803 and HGC-27 cells (Fig. 2d), while reduced NELFE expression dramatically inhibited the colony growth of BGC-823 and AGS cells (Fig. 2e). To verify whether these in vitro findings were relevant to GC tumor growth in vivo, a xenograft model of nude mice was generated by subcutaneous injection of GC cells with stable expression or knockdown of NELFE. Consistent with the in vitro observations, forced expression of NELFE substantially increased tumor size and tumor weight (Fig. 2f), while knockdown of NELFE led to opposite results (Fig. 2g). These findings revealed that NELFE plays important roles in GC cell growth in vitro and in vivo.

### Abnormal upregulation of NELFE is essential for GC cell migration, invasion and metastasis

**Fig. 3:**
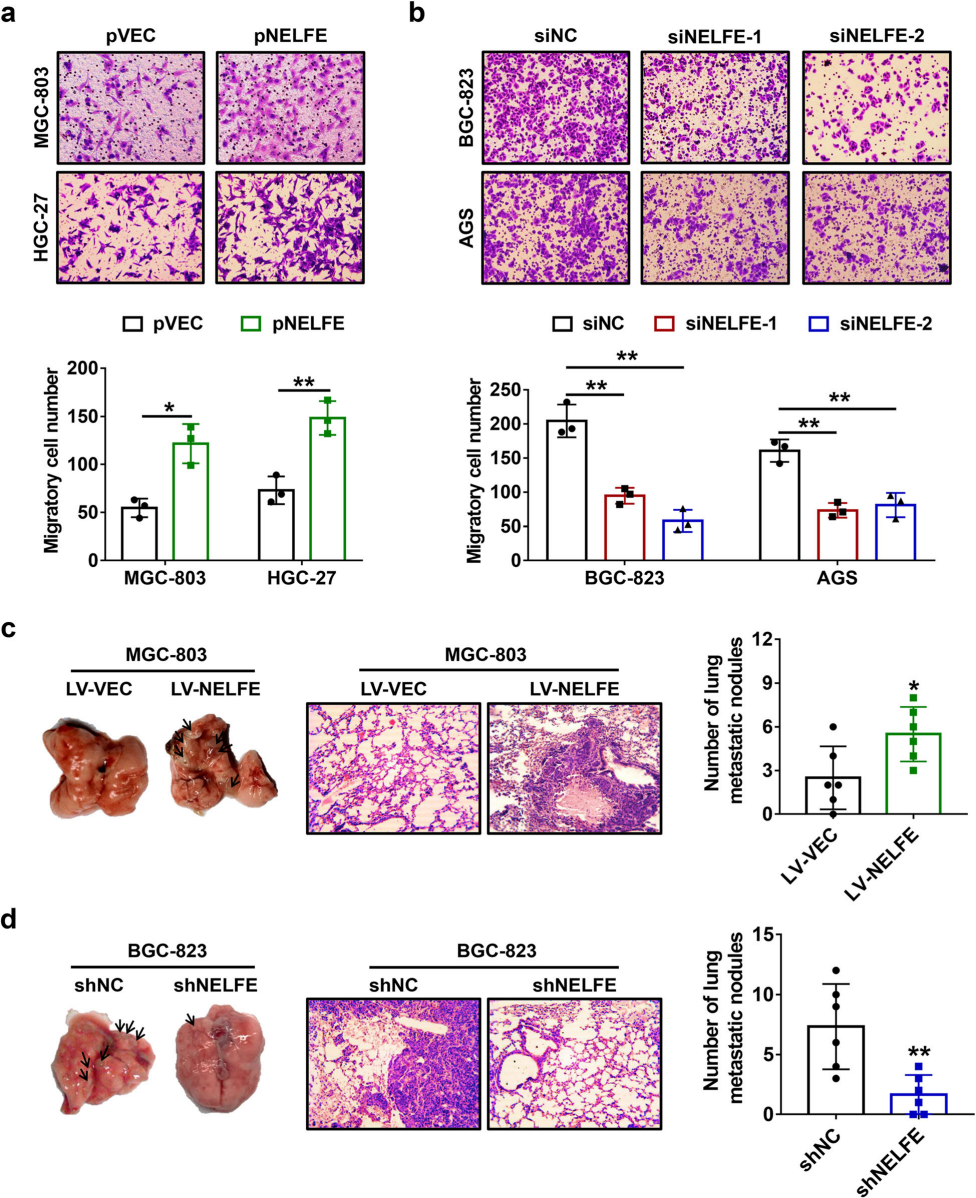
Positive effects of NELFE on GC cell migration in vitro and metastatis in vivo. **a-b** Transwell migration assays were carried out at 24 h after transfection of NELFE-expressing plasmids (**a**) or siRNAs (**b**), and representative images (upper) and corresponding statistical analysis of migratory cell number (bottom) were shown. Magnification: 100×. **c-d** A pulmonary metastasis model of nude mice was generated using cells with stable overexpression or knockdown of NELFE (*n*=6 per group). Representative lung tissues (left), HE staining images of the lung tissues (middle) and statistical analysis of the number of metastatic nodules on lung surface per mouse (right). *P < 0.05, **P < 0.01

Considering the IHC results that NELFE expression was significantly upregulated in metastatic tumor tissues in comparison with primary tumor tissues, we assumed that NELFE plays a role in tumor metastasis of GC. To verify this hypothesis, transwell migration and invasion assays were carried out in GC cells following transfection with pNELFE or siNELFE-1/2. The results showed that NELFE overexpression drastically increased the number of migratory cells (Fig. 3a), while siRNAs-mediated silencing of NELFE resulted in opposite results (Fig. 3b). As the same time, similar phenotypes were also observed in cell invasion assays (Supplementary Fig. 1a and b). Moreover, a lung metastasis model of athymic nude mice was generated using GC cells with stable overexpression or knockdown of NELFE. As expected, NELFE overexpression increased pulmonary metastatic nodules of MGC-803 cells (Fig. 3c), whereas NELFE knockdown in BGC-823 cells reduced pulmonary metastatic nodules compared with the control group (Fig. 3d). Collectively, the above findings suggested that NELFE acts as a facilitating factor for metastasis of GC.

### Oncogenic functions of NELFE in GC is mediated by CSNK2B

**Fig. 4:**
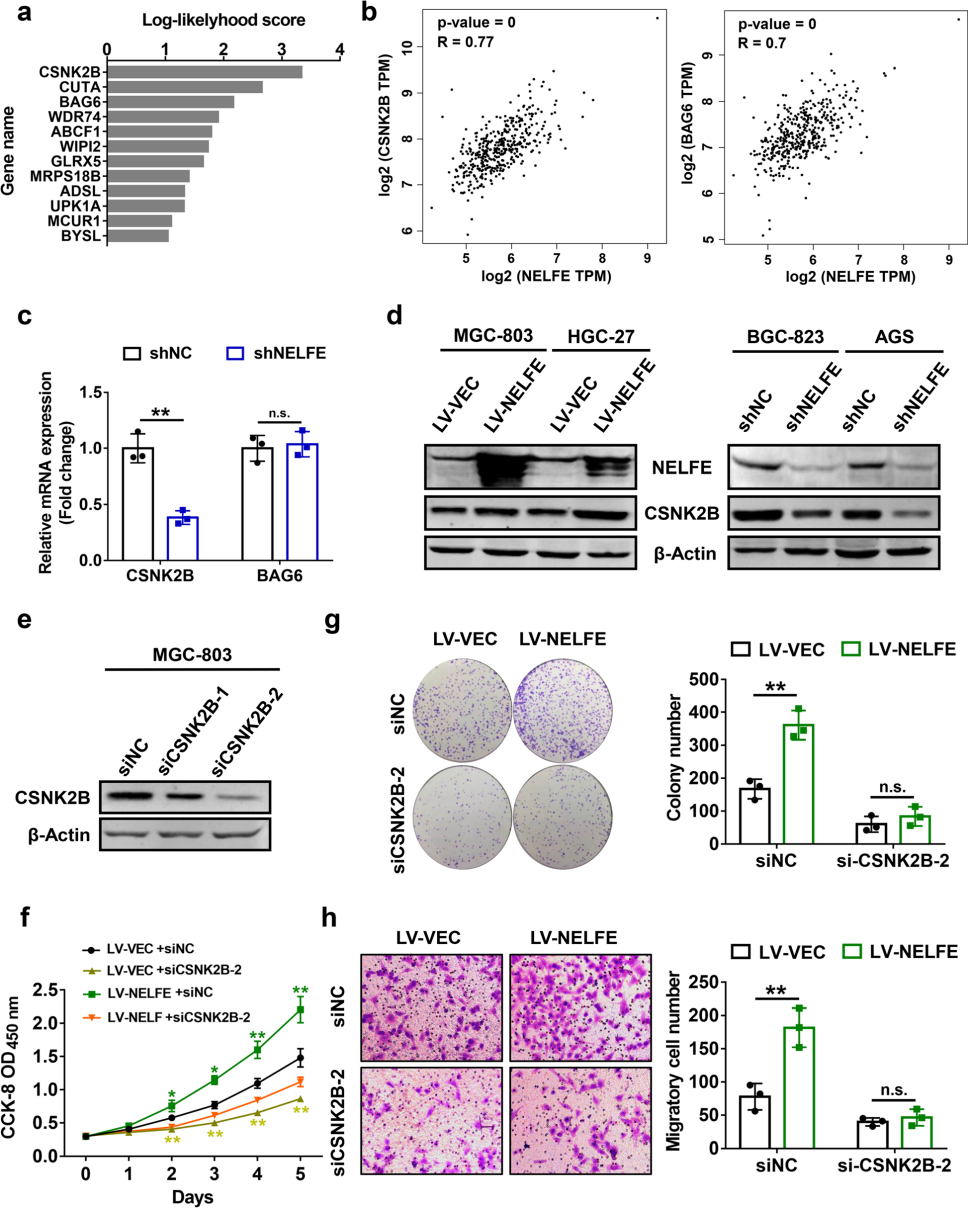
CSNK2B mediates the cancer-promoting functions of NELFE in GC. **a** Data from Coexpedia database (http://www.coexpedia.org/) was analyzed by informatics method to identify co-expressing genes of NELFE in GC, which were ranked by sum of their edges’ log-likelyhood scores. **b** RNA-sequencing data from TCGA database were analyzed using the online GEPIA tool (http://gepia.cancer-pku.cn/) to validate the results obtained from Coexpedia database, and CSNK2B and BAG6 were considered as candidate genes using the following criteria: Pearson correlation coefficient ≥0.7 and *P* value < 0.05. **c** CSNK2B and BAG6 mRNA levels in AGS cells with stable knockdown of NELFE were analyzed using qRT-PCR analysis. **d** Western blot analyses confirming the regulation of CSNK2B protein expression by NELFE in GC cells were shown. **e** After transfection with siRNAs against CSNK2B, cells were lysed and western blot analysis was performed to validate the knockdown efficacies. **f-h** CCK-8 assays (**f**), colony formation assays (**g**) and transwell migration assays (**h**) were carried out at 24 h after transfection of siNC or siCSNK2B-2 into MGC-803 cells with stable NELFE overexpression or the control cells, respectively. **P < 0.01, n.s. not statistically significant

To explore potential mechanisms possible for the cancer-promoting functions of NELFE, we performed gene co-expression analysis using data from Coexpedia database, and 12 co-expressed genes of NELFE were identified in GC (Fig. 4a). For further validation, Pearson correlation analysis was conducted to compare the relationships between NEFLE and these genes using an online Cancer Genome Atlas (TCGA) data visualization web tool GEPIA. Among the 12 genes, casein kinase 2 beta (CSNK2B, r = 0.77, *P* < 0.01) and BAG cochaperone 6 (BAG6, r = 0.70, *P* < 0.01) showed the highest correlation with NELFE in GC (Fig. 4b). Subsequently, we performed qRT-PCR analysis to determine their mRNA expression levels after knockdown of NELFE in AGS cells. Notably, a concomitant decrease in CSNK2B mRNA was observed, while BAG6 expression showed no significant change in AGS-shNELFE cells as compared to the control cells (Fig. 4c), indicating that CSNK2B was regulated by NELFE. Further western blot results confirmed the positive effects of NELFE on CSNK2B protein expression (Fig. 4d). CSNK2B is a regulatory subunit of casin kinase 2 (CK2) and plays a key role in regulating its kinase activity [20]. Previous studies have shown that CSNK2B is an oncogene in different human malignancies by directly trans-activating NF-κB signaling pathway [21, 22].

On the basis of these findings, we next synthesized two siRNAs against CSNK2B (siCSNK2B-1 and siCSNK2B-2) to test whether NELFE exerts its function dependent on CSNK2B expression. Western blot results showed that both siCSNK2B-1 and siCSNK2B-2 successfully silenced CSNK2B expression in MGC-803 cells, while siCSNK2B-2 exhibited a better knockdown efficiency than siCSNK2B-1 (Fig. 4e), thus siCSNK2B-2 was selected for further cell biology experiments. CCK-8 assays were carried out after transfection of siCSNK2B-2 into MGC-803 cells with NELFE overexpression. In line with previous reports [22–24], knockdown of CSNK2B significantly arrested cell proliferation, and more importantly, it also strongly weakened the positive effects of NELFE on proliferation ability of MGC-803 cells (Fig. 4f). Similar results were also observed in colony formation and transwell migration assays (Fig. 4g, h), hinting us that NELFE enhanced GC cell proliferation and migration through activating CSNK2B expression. Accordingly, it can be deduced that CSNK2B is a downstream factor of NELFE and mediates positive effects of NELFE on GC progression.

### NELFE enhances CSNK2B gene expression via activation of Wnt/β-catenin pathway

**Fig. 5:**
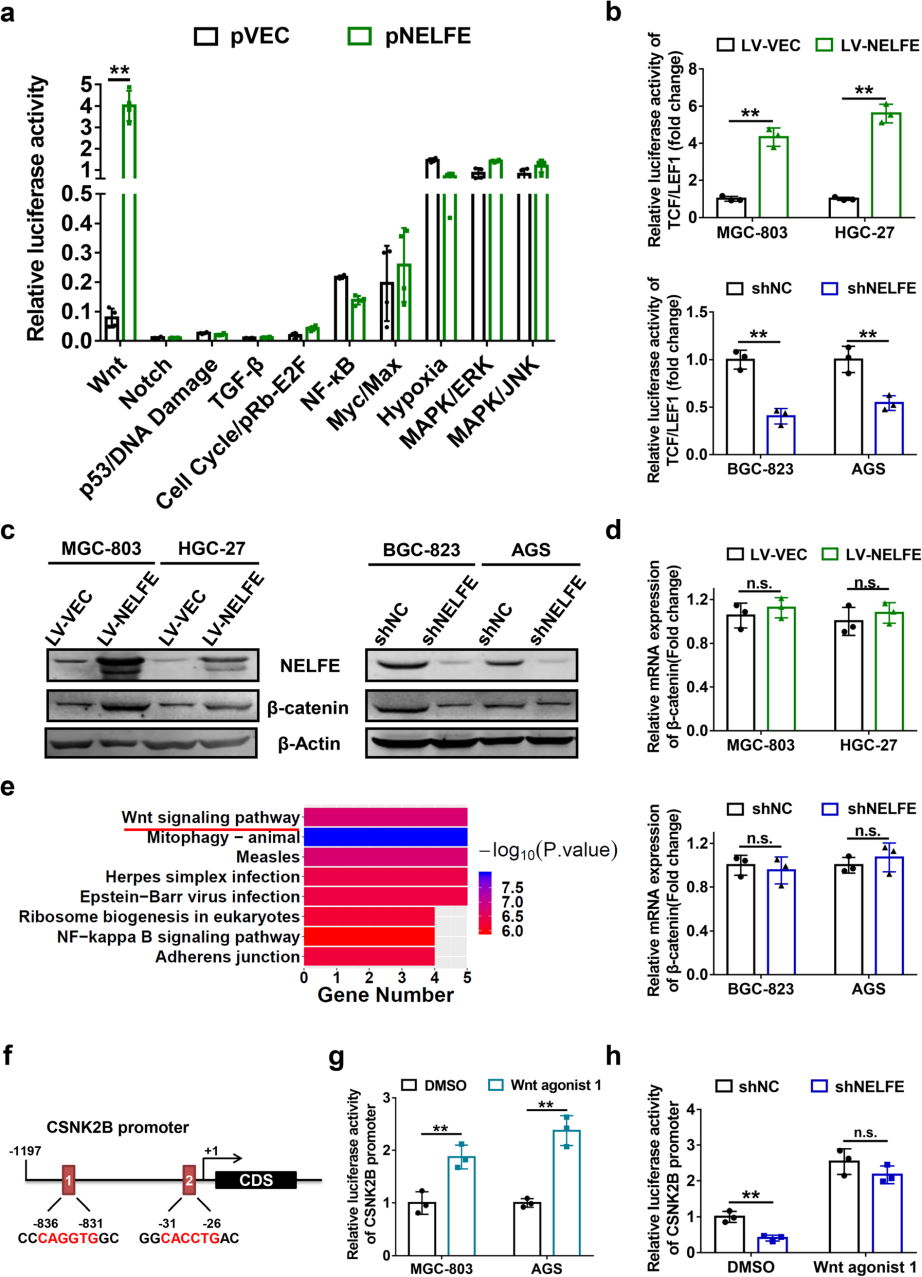
NELFE promotes CSNK2B expression via activating Wnt/β-catenin signaling pathway. **a** At 24 h after transfection of pNELFE into MGC-803 cells, Cignal Finder 10-Pathway Reporter Array was employed to uncover potential downstream signaling pathways regulated by NELFE. **b** The indicated cells were transiently co-transfected with the TCF/LEF1 firefly luciferase reporter construct and the Renilla luciferase vector for 24 h, and dual-luciferase reporter assays were conducted to detect relative TCF/LFE1 luciferase activity. **c-d** β-catenin (**c**) protein and mRNA (**d**) expression levels in the indicated GC cell lines were determined by western blot and qPCR analyses, respectively. **e** CSNK2B-interacting proteins were obtained from the STRING database at http://string-db.org, and Kyoto Encyclopedia of Genes and Genomes (KEGG) pathway enrichment analysis was performed by R programming language v3.5.2. **f** Schematic representation of two predicted TCF/LEF-binding sites in the promoter region of CSNK2B. **g** MGC-803 and AGS cells were transfected with CSNK2B promoter-driven luciferase constructs, 5 μM of Wnt agonist 1 was added into the cells concurrently, and CSNK2B promoter activity was measured using dual-luciferase assays after 24 h. **h** AGS cells with stable knockdown of NELFE and the control cells were treated with 5 μM of Wnt agonist 1, and DMSO were used as the control group. Then dual-luciferase reporter assays were performed to compare the difference of CSNK2B promoter activity between the two groups. **P < 0.01, n.s. not statistically significant

In an attempt to better understand how NELFE regulates CSNK2B expression in GC, we used the Cignal Finder 10-pathway reporter array to screen NELFE-associated signaling pathways in GC cells. Among the 10 signaling pathways directly related with tumor progression, Wnt/β-catenin pathway was strongly activated by NELFE overexpression (Fig. 5a). Next, a TCF/LEF1 luciferase reporter assay was performed to validate the effect of NELFE on the activity of canonical Wnt/β-catenin signaling pathway. Consistent with the data described above, higher levels of Wnt-dependent activities were observed in cells with NELFE overexpression, whereas silenced NELFE led to contrary results (Fig. 5b). Furthermore, upon NELFE overexpression or knockdown in GC cell lines, we examined the protein and mRNA level of β-catenin, a major component of Wnt signaling pathway [25], respectively. Interestingly, the protein level of β-catenin was enhanced by NELFE (Fig. 5c), while no significant change in mRNA level was observed after NELFE overexpression or knockdown (Fig. 5d), implying the regulation of β-catenin by NELFE is not at the transcriptional level. To explore whether CSNK2B is associated with Wnt/β-catenin signaling pathway, CSNK2B-interacting proteins were identified using the STRING database and further KEGG pathway analysis was performed. Notably, CSNK2B-interacting proteins were highly enriched in Wnt/β-catenin signaling pathway (Fig. 5e), suggesting a relationship between CSNK2B and Wnt signaling. Thereby, we aimed to explore whether Wnt/β-catenin signaling pathway could regulate CSNK2B expression in GC cells. By comparing sequences, 2 potential TCF/LEF response elements with the consensus sequence CANNTG (E-box motifs) were recognized in the promoter region of CSNK2B (Fig. 5f), indicating that CSNK2B might be a target gene of TCF/LEF transcription factors. To verify this possibility, CSNK2B promoter-luciferase reporter plasmid was constructed and a Wnt/β-catenin pathway activator (Wnt agonist 1) was used for dual-luciferase reporter assays. In agreement with our expectations, treatment with Wnt agonist 1 dramatically increased the activity of CSNK2B promoter in MGC-803 and AGS cells (Fig. 5g), indicating that Wnt/β-catenin pathway mediated transcriptional activation of CSNK2B gene. Further immunofluorescence analysis showed a significant enhancement of CSNK2B staining intensity after Wnt agonist 1 treatment (Supplementary Fig. 1c). Combined with the above analyses, we questioned whether NELFE regulated CSNK2B through activation of Wnt/β-catenin pathway. Dual luciferase assays demonstrated that NELFE inhibition significantly reduced CSNK2B promoter activity (*P* < 0.01), which could be rescued by treatment of Wnt agonist 1 (Fig. 5h). Collectively, these data strongly suggested that Wnt/β-catenin pathway mediate the activation of CSNK2B expression induced by NELFE.

### Increased expression of NELFE, β-catenin and CSNK2B in GC tissues are positively correlated with each other

**Fig. 6:**
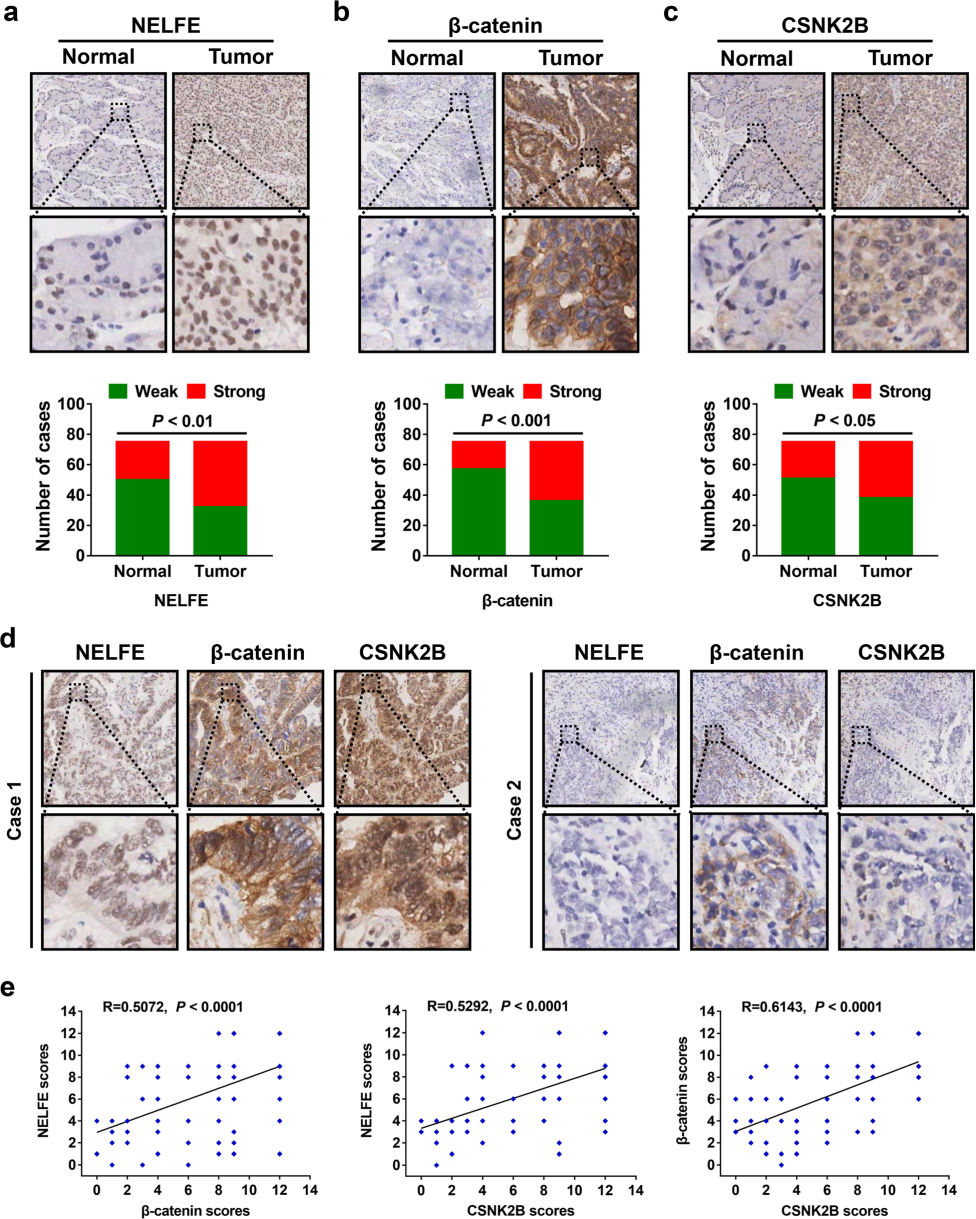
Overexpressed NELFE, β-catenin and CSNK2B in GC are positively correlated with each other. **a-c** Representative IHC images of NELFE (**a**), β-catenin (**b**) and CSNK2B (**c**) in GC tumor tissues and adjacent normal tissues. Magnification: 40× (upper) and 200× (bottom). Bar graphs showed the statistical analysis of three proteins expression between tumor tissues and adjacent normal tissues. **d** Representative IHC images of NELFE, β-catenin and CSNK2B at the same location on serial tumor sections. **e** The correlation among these three genes in GC tumor tissues were analyzed using Pearson’s correlation coefficient analysis (*n*=75) according to their respective IHC scores, and some of the dots represented more than one specimen

To investigate possible clinical relevance of these 3 genes in GC, we performed an IHC analysis using consecutive sections on a TMA containing 75 paired tumor/adjacent normal tissues from GC patients. Consistent with the IHC data described above, there was a significant increase in NELFE expression in tumor tissues than in adjacent normal tissues (*P* < 0.01, Fig. 6a), and the same trend was observed in β-catenin expression (*P* < 0.001, Fig. 6b), which has been well understood in previous studies [26, 27]. Similarly, high CSNK2B expression was also found in tumor tissues when compared with adjacent normal tissues (*P* < 0.05, Fig. 6c). Further analysis of the same locations on consecutive tumor tissues sections indicated a consistent trend among the expression levels of all three genes in GC (Fig. 6d). Moreover, Pearson correlation analyses were applied based on the IHC scores of NELFE, β-catenin and CSNK2B (Fig. 6e). As expected, both β-catenin (R = 0.5072, *P* < 0.0001) and CSNK2B (R = 0.5292, *P* < 0.0001) expression showed a strong correlation with NELFE expression in GC, and both of them were positively associated with each other (R = 0.6143, *P* < 0.0001). Thus, these findings further supported the regulatory relationship among NELFE, β-catenin and CSNK2B in GC.

## Discussion

It is well established that tumorigenesis is a complex process commonly accompanied with aberrant activation of oncogenic genes [28]. NELFE, a member of RNA-binding proteins (RBP) which is involved in regulating transcription elongation and RNA homeostasis, has received increasing attention in recent years in the development of human malignancies for its key roles in dysregulated transcriptional events [29–31]. Nevertheless up to date, the relationship between NELFE and GC progression remains largely unknown. In the present study, we for the first time investigated the expression features of NELFE in GC. We found NELFE was dramatically overexpressed in GC tumor tissues than in adjacent normal tissues by IHC and qPCR analysis, and metastatic tumors exhibited an increased NELFE expression than primary tumors, indicating the participation of NELFE in GC progression. Gene expression data from GEO database further confirmed these results, and a negative correlation of NELFE expression with the overall and first progression survival rates of GC patients were found based on the online survival database Kaplan-Meier plotter. Through gain- or loss-of-function experiments, we showed that NELFE facilitated GC cell proliferation, migration and invasion in vitro, and further animal experiments confirmed this conclusion in vivo. These observations unambiguously supported the cancer-promoting role of NELFE in GC, consistent with previous studies on NELFE in HCC or pancreatic cancer [12, 30].

In mechanistic studies, we performed comprehensive analysis combining the bioinformatics data and our experimental evidence to elucidate the exact mechanisms by which NELFE exerts its effects during GC tumorigenesis, and CSNK2B, a regulatory subunit of casein kinase II (CK2), was identified as a downstream effector of NELFE. Studies have demonstrated that CSNK2B is essential for the kinase activity of CK2, and overexpression of CSNK2B has been reported to promote HCC tumorigenesis via trans-activation of NF-κB [20, 21]. Although there is currently no available evidence regarding the roles of CSNK2B in GC, our cell biology experiments confirmed that CSNK2B facilitated GC cell proliferation and migration, and it also mediated the pro-oncogenic functions of NELFE in GC.

Another important finding of our work is that Wnt/β-catenin signaling pathway was uncovered to mediate the regulation of NELFE on CSNK2B expression. As we known, activation of β-catenin is then able to translocate into the nucleus and form a complex with T-cell factor/lymphoid enhancer factor (TCF/LEF) proteins to induce the transcription of genes involved in cell growth and proliferation, such as c-myc and cyclin D1 [32]. Consistently, dual-luciferase assays showed that CSNK2B gene was transcriptionally activated by TCF/LEF, which means Wnt/β-catenin signaling pathway is a positive regulator of CSNK2B expression. Therefore, these findings identified a NELFE/β-catenin/CSNK2B axis in promoting the initiation and development of GC. However, the exact mechanism for how NELFE enhanced the protein expression of β-catenin, still needs more in-depth studies. In addition, it is worth noting that a recent study reported that NELFE is able to decrease the stabilization of N-Myc downstream-regulated gene 2 (NDRG2), which is involved in the inhibition of the Wnt/β-catenin signaling pathway [30], thus providing a possible explanation for the effects of NELFE on β-catenin expression. Certainly, more experimental evidence is needed to verify this hypothesis. Finally, our IHC analysis on consecutive sections of a TMA confirmed that NELFE, β-catenin and CSNK2B expression were all highly expressed in tumor tissues as compared to adjacent normal tissues, and all these three genes expression in GC were positively correlated with each other, which provides further support to the above experimental findings.

## Conclusion

**Fig. 7:**
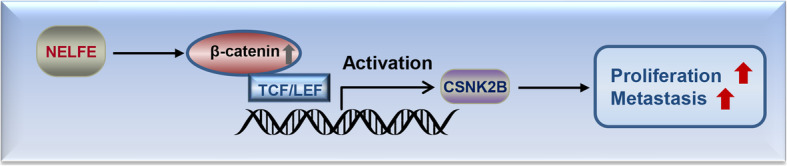
Schematic illustration of the NELFE/β-catenin/CSNK2B signaling axis in GC progression. Increased NELFE expression activates the Wnt/β-catenin pathway via upregulation of β-catenin. In the nucleus, interaction of β-catenin with TCF/LEF transcription factors results in activation of CSNK2B expression, which finally promotes GC cell proliferation and metastasis

This study revealed that NELFE functions as an oncogene in GC and a new oncogenic NELFE/β-catenin/CSNK2B signaling axis was identified, which may help to develop new diagnosis and treatment strategies against GC (Fig. 7).

## Supplementary Information

**Additional file 1: Supplementary Table 1.** Primer sequences for qRT-PCR analysis.

**Additional file 2: Supplementary Figure 1.** a-b Cell invasion assays were carried out at 24 h after transfection of NELFE-expressing plasmids (a) or siRNAs (b), and representative images (upper) and quantification analysis (bottom) were shown. Magnification: 100×. *P < 0.05, **P < 0.01. **c** Representative images of CSNK2B immunofluorescence staining (red) in MGC-803 cells after Wnt agonist 1 treatment (5 μM). Magnification: 40×.

## Data Availability

All data generated or analyzed during this study are included in this published article and its supplementary information files.

## Abbreviations

NELFE: Negative elongation factor complex member E
GC: Gastric cancer
IHC: Immunohistochemistry
qPCR: Quantitative real-time PCR
TMA: Tissue microarray
RNAPII: RNA polymerase II
NELF: Negative elongation factor
DRB: 5,6-dichloro-1β-D-ribofuranosylbenzimidazole
DSIF: DRB-sensitivity-inducing factor
RNM: RNA recognition motif
HCC: Hepatocellular carcinoma
CCK-8: Cell Counting Kit-8
OD: Optical density
PBST: PBS containing 0.05% Tween-20
H&E: Hematoxylin and eosin
ANOVA: One-way analysis of variance
SD: Standard deviation
GEO: Gene Expression Omnibus
BAG6: BAG cochaperone 6
CSNK2B: Casein kinase 2 beta
TCGA: Cancer Genome Atlas
RBP: RNA-binding proteins
CK2: Casein kinase II
TCF/LEF: T-cell factor/lymphoid enhancer factor
NDRG2: N-Myc downstream-regulated gene 2
