# Supplementary material for: Overexpression of NELFE contributes to gastric cancer progression via Wnt/β-catenin signaling-mediated activation of CSNK2B expression
Source: J Exp Clin Cancer Res. 2021 Feb 1;40:54. doi: 10.1186/s13046-021-01848-3 (PMC7851912; doi:10.1186/s13046-021-01848-3)
Supplement: Supplementary file 1 — Additional file 1: Supplementary Table 1. Primer sequences for qRT-PCR analysis. [file 13046_2021_1848_MOESM1_ESM.docx]

**Supplementary Table 1 Primer sequences for qRT-PCR analysis**

| Gene | Forward (5’ to 3’) | Reverse (5’ to 3’) |
| --- | --- | --- |
| NELFE | GAGGGGAAGTTAAAGGACC | GACACAAAGCTCTCATACAG |
| CSNK2B | TGAGCAGGTCCCTCACTACC | GTAGCGGGCGTGGATCAAT |
| β-catenin | TTGAAAATCCAGCGTGGACA | TCGAGTCATTGCATACTGTC |
| BAG6 | ACAACGGCTCATTTACCAGGG | CCAGGTGGATAACCTTTCCCC |
| β-actin | CCTGGCACCCAGCACAATG | GGGCCGGACTCGTCATACT |
