# Supplementary figures and images for: Overexpression of NELFE contributes to gastric cancer progression via Wnt/β-catenin signaling-mediated activation of CSNK2B expression
Source: J Exp Clin Cancer Res. 2021 Feb 1;40:54. doi: 10.1186/s13046-021-01848-3 (PMC7851912; doi:10.1186/s13046-021-01848-3)

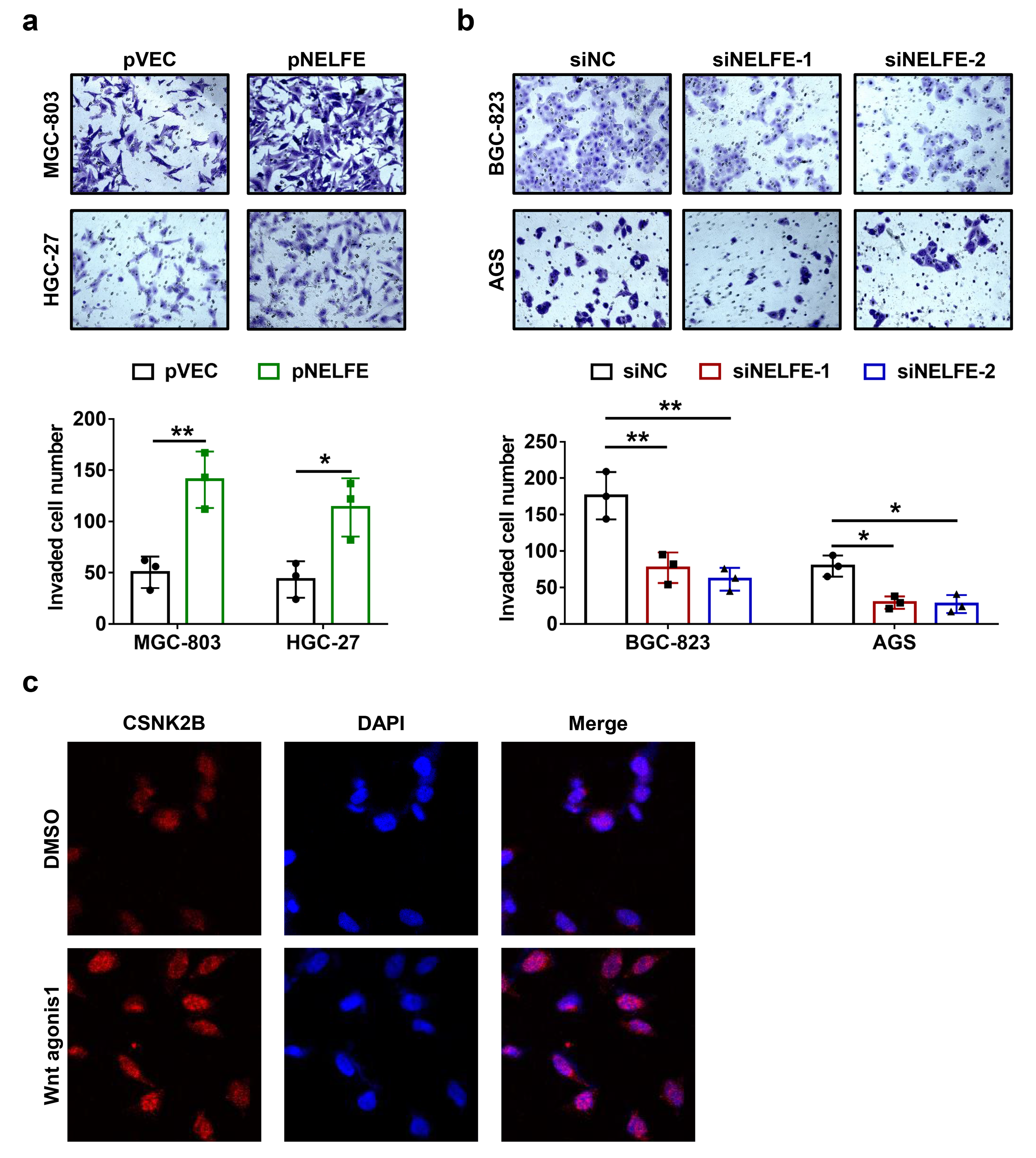

Supplement: Supplementary file 2 — Additional file 2: Supplementary Figure 1. a-b Cell invasion assays were carried out at 24 h after transfection of NELFE-expressing plasmids (a) or siRNAs (b), and representative images (upper) and quantification analysis (bottom) were shown. Magnification: 100×. *P < 0.05, **P < 0.01. c Representative images of CSNK2B immunofluorescence staining (red) in MGC-803 cells after Wnt agonist 1 treatment (5 μM). Magnification: 40×. [file 13046_2021_1848_MOESM2_ESM.tif]
